# Genomic profiling of bovine corpus luteum maturation

**DOI:** 10.1371/journal.pone.0194456

**Published:** 2018-03-28

**Authors:** Sigal Kfir, Raghavendra Basavaraja, Noa Wigoda, Shifra Ben-Dor, Irit Orr, Rina Meidan

**Affiliations:** 1 Department of Animal Sciences, The Robert H. Smith Faculty of Agriculture, Food and Environment, Hebrew University of Jerusalem, Rehovot, Israel; 2 Bioinformatics unit, Department of Life Sciences Core Facilities, Weizmann Institute of Science, Rehovot, Israel; Universite Clermont Auvergne, FRANCE

## Abstract

To unveil novel global changes associated with corpus luteum (CL) maturation, we analyzed transcriptome data for the bovine CL on days 4 and 11, representing the developing vs. mature gland. Our analyses revealed 681 differentially expressed genes (363 and 318 on day 4 and 11, respectively), with ≥2 fold change and FDR of <5%. Different gene ontology (GO) categories were represented prominently in transcriptome data at these stages (e.g. days 4: cell cycle, chromosome, DNA metabolic process and replication and on day 11: immune response; lipid metabolic process and complement activation). Based on bioinformatic analyses, select genes expression in day 4 and 11 CL was validated with quantitative real-time PCR. Cell specific expression was also determined in enriched luteal endothelial and steroidogenic cells. Genes related to the angiogenic process such as *NOS3*, which maintains dilated vessels and *MMP9*, matrix degrading enzyme, were higher on day 4. Importantly, our data suggests day 11 CL acquire mechanisms to prevent blood vessel sprouting and promote their maturation by expressing *NOTCH4* and *JAG1*, greatly enriched in luteal endothelial cells. Another endothelial specific gene, *CD300LG*, was identified here in the CL for the first time. *CD300LG* is an adhesion molecule enabling lymphocyte migration, its higher levels at mid cycle are expected to support the transmigration of immune cells into the CL at this stage. Together with steroidogenic genes, most of the genes regulating de-novo cholesterol biosynthetic pathway (e.g *HMGCS*, *HMGCR*) and cholesterol uptake from plasma (*LDLR*, *APOD* and *APOE*) were upregulated in the mature CL. These findings provide new insight of the processes involved in CL maturation including blood vessel growth and stabilization, leucocyte transmigration as well as progesterone synthesis as the CL matures.

## Introduction

The ovulatory surge of gonadotropins triggers extensive structural, cellular, and molecular changes in the preovulatory follicle, leading to ovulation and corpus luteum (CL) formation [1, 2]. The new CL develops from cells that remain in the follicular wall following ovulation but is eventually composed of multiple, distinctive cell types including luteal steroidogenic cells (LSC; small and large cells that originate from theca and granulosa, respectively) and non-steroidogenic cells (luteal endothelial cells—LEC; pericytes, fibrocytes, and immune cells) [3–6]. Various immune cells such as lymphocytes, macrophages, neutrophils, eosinophils, and dendritic cells are identified in the mature CL [7–11]. Yet, the molecules responsible for this massive infiltration of immune cells, much before luteolysis, are still poorly characterized. Small and large luteal cells have distinct characteristics [12, 13] but they are both engaged in progesterone production. Progesterone dramatically increases after the LH surge and CL formation reaching a plateau at the mid luteal stage that is maintained until days 15–16 of the cycle [14]. Cholesterol is the precursor for steroid hormone synthesis including progesterone; it can be derived from the diet through low-density lipoprotein receptor (LDLR) mediated uptake or is synthesized de novo [15, 16], but only few studies examined cholesterol synthesis in the CL [17–19] Along with increased steroidogenesis in the developing CL, robust angiogenesis takes place resulting in a highly vascular gland with LEC comprising the larger part of its cells, more than 50% [20–22]. The development of an elaborate network of blood vessels in the CL endows this gland with one of the highest blood flow per unit mass in the body [23], which guarantees the necessary supply of nutrients and hormones allowing for its proper function. The short period of angiogenesis is later followed by maintenance and stabilization of the vasculature in the fully active CL. The information described above is derived from numerous studies analyzing morphometric, biochemical and molecular changes associated with the maturation of the CL [5, 13, 24].

To unveil novel regulatory mechanisms and gain more comprehensive knowledge of the changes that accompany CL maturation, we analyzed transcriptome data for the bovine CL on early vs mature CL (days 4 and 11, respectively). Based on bioinformatic analyses, the profile of select gene expression in these CL and in isolated luteal cell types (endothelial and steroidogenic) was validated with quantitative real-time PCR (qPCR).

## Materials and methods

### Bioinformatic analyses

Our previously published dataset (accession no. GSE23348; Gene Expression Omnibus) was re-analyzed in order to compare the transcriptomes of early and mid-stage bovine corpora lutea. Microarray analysis was performed using the PartekGenomics Suite, version 6.5, 2010 (http://www.partek.com). Data was normalized and summarized using the robust multi-average method, followed by analysis of variance (ANOVA) for differentially expressed genes (DEG). Cluster analysis of the DEG (cutoffs: p-value ≤ 0.05 and absolute fold-change ≥ 2) was also performed. GO (Gene Ontology) analysis of the DEG was performed using the DAVID online application: https://david.ncifcrf.gov/. In order to obtain the most meaningful clusters, the threshold of EASE score (a modified Fisher exact p-value) for gene enrichment analysis was set to ≤ 0.05. The p-values were corrected for multiple comparisons using the Benjamini and Hochberg [25] method. An additional GO analysis was performed with Ontologizer 2.0 [26], using the term-for-term algorithm. (Term-for-Term), GO annotation files (gene_association.goa_ref_cow.gz and gene_association.goa_cow.g) were downloaded from http://beta.geneontology.org/page/download-mappings in October 2014. Ontologizer recognized 19,930 gene symbols from the background and 336 and 294 gene symbols of up- and down-regulated (p < 0.05; fold-change ≥ 2, Benjamini–Hochberg <5%) gene sets, respectively. For additional functional analysis we used Ingenuity pathway analysis (IPA): A list of DEG with associated fold change values (fold change |≥ 2|) was uploaded into the IPA server (http://www.ingenuity.com/). Using information stored in the Ingenuity Knowledge Base (IKB), genes were mapped to networks and pathways. The significance of the association between genes/networks/pathways was evaluated by right-tailed Fischer’s exact test (p<0.05). The IPA networks are ranked by a score derived from a p-value and indicates the likelihood of the focus genes appearance in a network, computed as p-score = -log10 (p-value).

If not otherwise mentioned biochemical were from Sigma-Aldrich Israel Ltd and tissue culture materials were from Biological Industries, Kibbutz Beit Haemeek, Israel.

### Animals and CL collection

All animal procedures were approved by the All University Committee on Animal Use and Care at Michigan State University. Thirty Angus crossbred heifers (average body wt 395 ± 7 kg) were synchronized for estrus with two 25-mg injections of PG (Lutalyse; Pfizer, Kalamazoo, MI) 11 days apart. Heifers were monitored four times daily for behavioral estrus (day 0), and ovulation was verified by transrectal ultrasonography (Aloka 500V with a 7.5-MHz linear transducer; Aloka, Wallingford, CT) on day 1. CL collection was described in detail in our previous publication [27, 28]. Briefly, CL were collected after epidural anesthesia on Day 4 or Day 11 (n = 5 for each stage) of the estrous cycle. CL were diced and snap frozen in liquid nitrogen and stored at −80°C until RNA extraction with RNAeasy kits. Total RNA was subjected to reverse transcription for synthesis of cDNA, which served as a template for synthesis of full-length biotin labeled cRNA with the GeneChip HT One-Cycle Target Labeling and Controls Kit (Affymetrix, Santa Clara, CA.).

### Isolation and culture of luteal cells

Luteal cells were dispersed and enriched as previously described [27, 29, 30]. Briefly, corpora lutea at the mid-luteal phase (day 9–14) were dispersed using collagenase. Dispersed cells were suspended in 1% BSA in M-199, mixed with magnetic tosyl activated beads pre-coated with BS-1 lectin (0.15 mg/ml) from bovine endothelial cells and incubated for 25 min at 4 °C on a rocking platform. The adherent cells (BS-1 positive) were washed with M-199 containing 1% BSA and concentrated using a magnet until the supernatant was free of BS-1 negative cells. The adherent cells were subsequently eluted by 0.2 M lactose solution in PBS. Freshly isolated BS-1-positive cells (enriched LEC) and non-adherent cells (BS-1 negative-enriched luteal steroidogenic cells; LSC) were collected for RNA extraction.

### Isolation and culture of granulosa cells

Bovine granulosa cells were isolated from ovaries collected at a local slaughterhouse as previously described [12]. Only large follicles (>10 mm in diameter) containing ≥ 4 million viable cells were used. Granulosa cells were enzymatically dispersed using a mixture of collagenase type IA (5000 units), hyaluronidase III (1440 units), DNase I (390 units), and cultured overnight in DMEM/F12 containing 3% fetal calf serum (FCS), 2mM L-glutamine, penicillin (100U/ml) /streptomycin (1mg/ml) solution.

### RNA extraction and qRT-PCR

Total RNA was isolated from enriched luteal cells and granulosa cells using TriFast reagent (Peqlab Biotechnologie GmbH) according to the manufacturer’s instructions. 1μg of total RNA was reverse transcribed using M-MuLV Reverse Transcriptase (200units/μl), M-MuLV RT Buffer (New England Biolabs, Ipswich, MA, USA), random primer (100nM), oligo-dT (100 μM) and dNTPs mix (100 mM) (Bioline Reagents Limited, London, UK). qRT-PCR were performed using the LightCycler 96 SW 1.1 software (Roche Diagnostics Corporation, Indianapolis, IN, USA) and Platinum SYBR Green (SuperMix; Invitrogen) as previously described [31]. Gene expression (*NOTCH4*, *MMP9*, *JAG1*, *CD300LG*, *HMGCS1*, *HMGCR*, *SC5DL*, and *NOS3*) was analyzed by qRT-PCR. The Ribosomal Protein S26 (*RPS26*) gene was used as the normalizing gene. Sequences of primers used for qRT-PCR are listed in Table 1. All primers were designed to have single-product melting curves, as well as consistent amplification efficiencies of >96% [32]. The threshold cycle number (Ct) was used to quantify the relative abundance of the gene; arbitrary units were calculated as 2^−ΔCt^ = 2^− (Ct target gene−Ct housekeeping gene)^.

**Table 1:** Genes, sequences and Genbank accessions of the primers used in qPCR.

Gene symbol	Primer	Sequence	Accession #
***CD300LG***	Forward	*gatgaagagcccggcctct*	XM_002696037
	Reverse	*cttgtgctcccaggttacg*	
***HMGCS1***	Forward	*agctcttgggatggacggtatg*	NM_001206578
	Reverse	*cggctccaactccacctgta*	
***SC5DL***	Forward	*tcccgttacacaagacatcctgg*	NM_001035356
	Reverse	*cgtgcatcctgtacacgtggctg*	
***HMGCR***	Forward	*tggcatcacctgacctggac*	NM_001105613
	Reverse	*tggcttgagacgcctgaagg*	
***MMP9***	Forward	*gagagggtcgcaatgatg*	NM_174744
	Reverse	*ctggcacggaggtgtgatcta*	
***JAG1***	Forward	*tcctacactttgctcgtggag*	NM_001191178
	Reverse	*acttattgcagccgaagcc*	
***NOTCH4***	Forward	*caggccatctctgtgaaattc*	NM_001206948
	Reverse	*ggtggcaggtgcagttgtctt*	
***NOS3***	Forward	*cctcaccgctacaatatcct*	NM_181037
	Reverse	*tgctcgttgtccaggtgcttc*	
***RPS26***	Forward	*ccaactgtgcccgatgtg*	NM_001015561
	Reverse	*ttcccgagagcgattcctga*	

### Statistical analyses

All statistical analyses were conducted using GraphPad Prism version 4.03 Software (GraphPad Software). Data are presented as the means of ±S.E.M; cell culture experiments were repeated at least three times; Asterisks represent significant differences between groups. *P<0.05, **P<0.01, ***P<0.001. Additional information is provided in figure legends.

## Results

### Identification of differentially expressed genes and functional analyses in day 4 and day 11 CL

**Fig 1:**
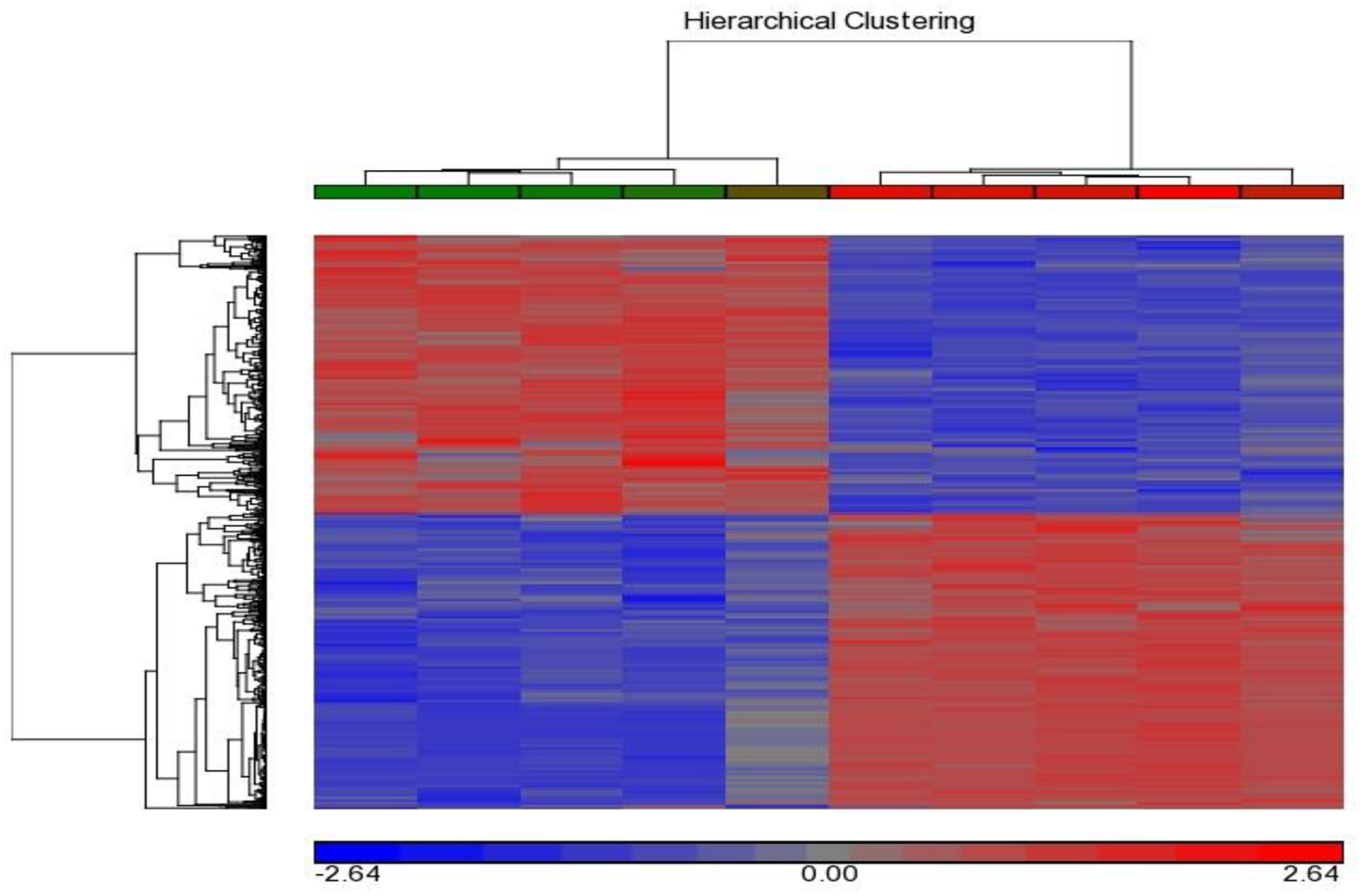
Clustering of bovine CL DEG on day 4 vs. day 11 using hierarchical clustering performed with Partek Genomics Suite. The Hierarchical clustering of 681 expressed genes (fold-change |≥ 2|, FDR of <5%) in day 4 and day 11 CL. Each column represents an Affymetrix chip (n = 10) and each row represents a gene. The deep red color represents relative upregulated expression, while the deep blue color represents relative down regulated expression.

To identify the gene expression that differ in the early (day 4) and mid-luteal (day 11) stages we reanalyzed a previously described microarray dataset [27, 28]. Initially hierarchical clustering (Fig 1) of the microarray results was carried out using the Partek Genomics Suite. High uniformity was displayed between the Affymetrix chips interrogated with samples within each time point (n = 5 per day). 681 DEG, with |≥ 2| fold change and FDR of <5% were found. About half of these genes (363) were expressed at a higher level in the early luteal stage compared to the mid luteal stage, while 318 genes had a lower expression level in the early luteal stage (day 4 of the cycle) as compared to the mid luteal stage (day 11 of the cycle).

**Fig 2:**
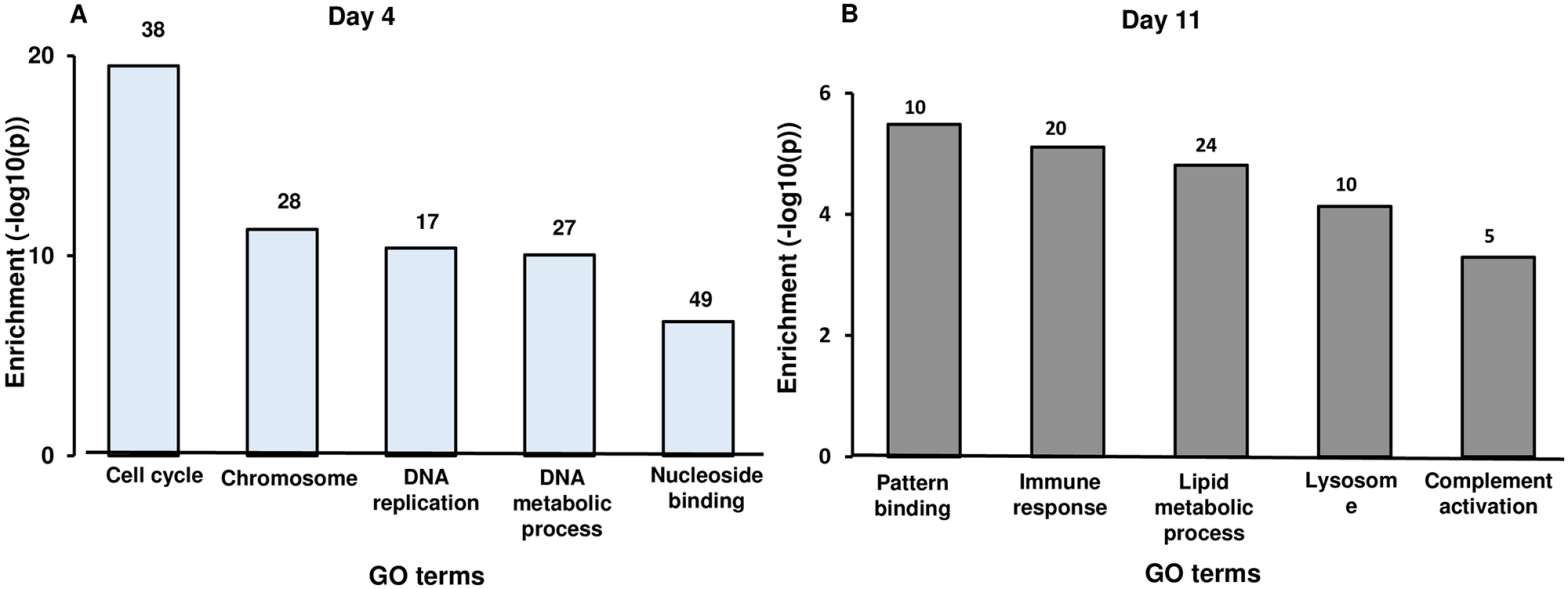
Enriched Gene-Ontology (GO terms) in day 4 vs. day 11 bovine CL. GO term analysis (using DAVID) of DEG (fold-change |≥ 2|, FDR of <5%) was carried out. The X-axis are selected GO terms and the Y-axis are −log10 (p-value) enrichment score of the GO terms. The numbers above bars are the numbers of DEG assigned to each GO terms.

Next, we performed functional enrichment analysis using David clustering and Ontologizer 2.0 tools on each gene list (day 4 and day 11). DEG were classified into 70 and 37 enriched GO terms in the early and mid-luteal stages, respectively (S1 File). On day 4, the cluster enriched at the highest significance level, including 38 genes (>10% of the tested genes), was cell cycle (GO: 0007049) with a p-value of 3.10 e-20 (all p-values presented here are after correction according to Benjamini and Hochberg [25]). Other selected clusters that were significantly enriched in day 4 CL data were: chromosome (GO: 0005694), DNA metabolic process (GO: 0006259), nucleoside binding (GO: 0001882) and DNA replication (GO: 0006260). Genes with higher mRNA abundance in day 11 CL were clustered into the following GO terms: pattern binding (GO: 0001871), immune response (GO: 0006955), lipid metabolic process (GO: 0006629), lysosome (GO: 0005764) and complement activation (GO: 0006956) (Fig 2).

**Fig 3:**
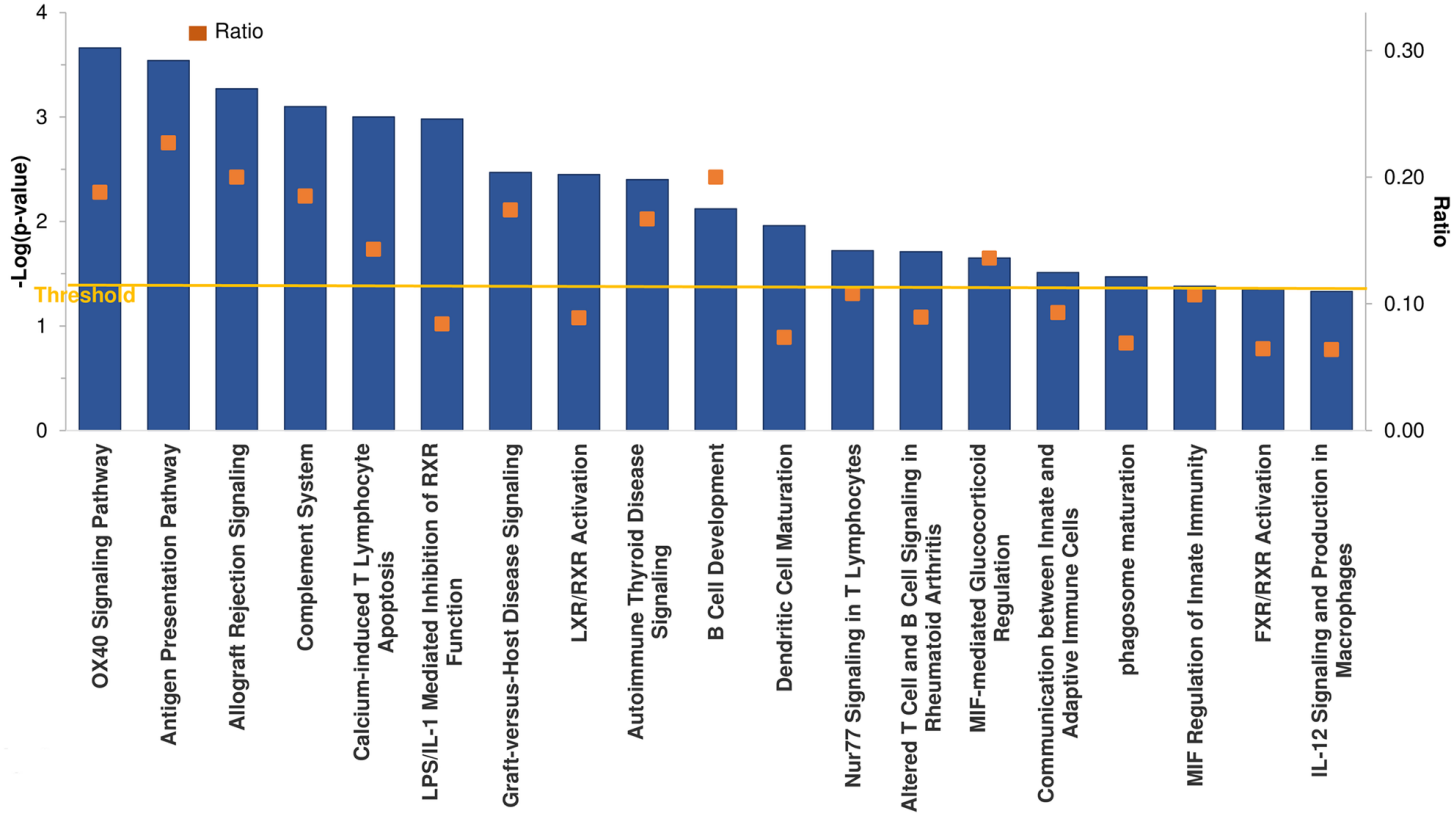
Up-regulated immune canonical pathways in day 11 CL, performed using Ingenuity Pathway Analysis (IPA) for immune pathways in day 11 vs. day 4 CL. The vertical axis (left) shows the −log of the p-value calculated based on Fisher’s exact test. The ratio (vertical axis, right) is calculated by the number of genes in a given pathway that meet cutoff criteria, divided by the total number of genes that make up that pathway. The orange line stands for the threshold above which there are statistically significantly values (by default P<0.05).

Pathway analysis of array data was performed using IPA software. From the 318 DEG identified in day 11 CL, 41 significant (p< 0.05) canonical pathways were found; ~50% were immune related. They included pathways such as the OX40 (member of the TNF receptor family, expressed on activated CD4+ T cells and CD8+ T cells) signaling pathway, antigen presentation pathway, B cell development, complement system, and dendritic cell maturation (Fig 3).

**Fig 4:**
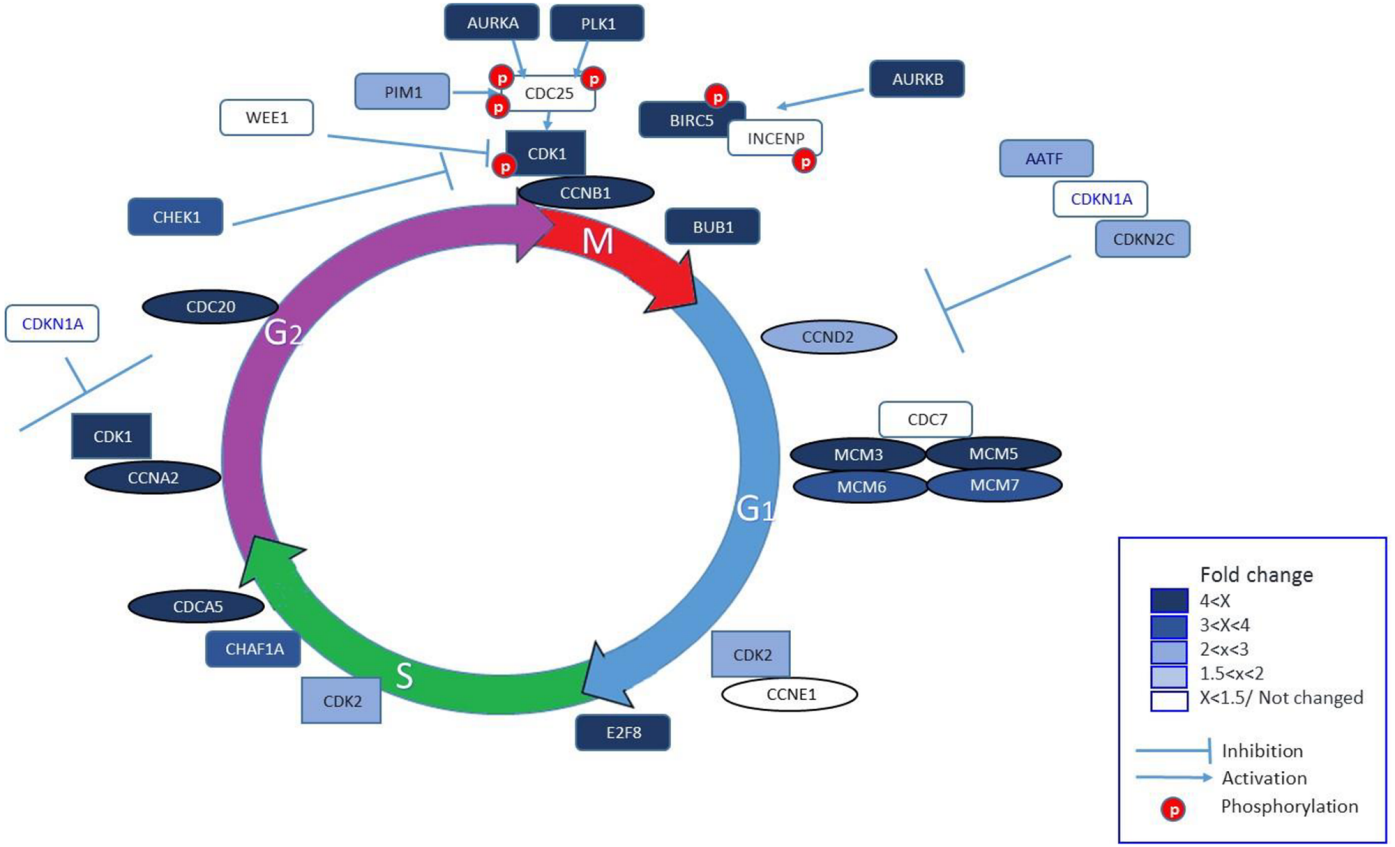
Illustration of differentially expressed cell cycle genes in early CL. The diagram depicts up-regulated genes that were differentially expressed (p<0.05) in day 4 vs. 11 CL. Genes appear in the diagram in relation to cell cycle steps. Intensity of the shading increases with the magnitude of the change (see inset).

To demonstrate DEG related to cell cycle of greater abundance in day 4 CL, selected genes that were significantly upregulated using Ontologizer 2.0 (fold change >2; p<0.05) were marked on cell cycle diagram and colored according to their fold change (Fig 4). It shows genes involved in the G1/S transition, progression through S, G2 and M- phases. Genes include cyclins and their CDKs (Cyclin-dependent kinases), CDKs inhibitors, MCMs (mini-chromosome maintenances), CDCs (cell division cycles), cell cycle associated kinases (PLK1, AURKA, AURKB, PIM1), and survival/apoptosis factors (BIRC5/survivin, AAFT) (Fig 4).

### Pattern of endothelial/blood vessel gene expression in day 4 and 11 CL

**Fig 5:**
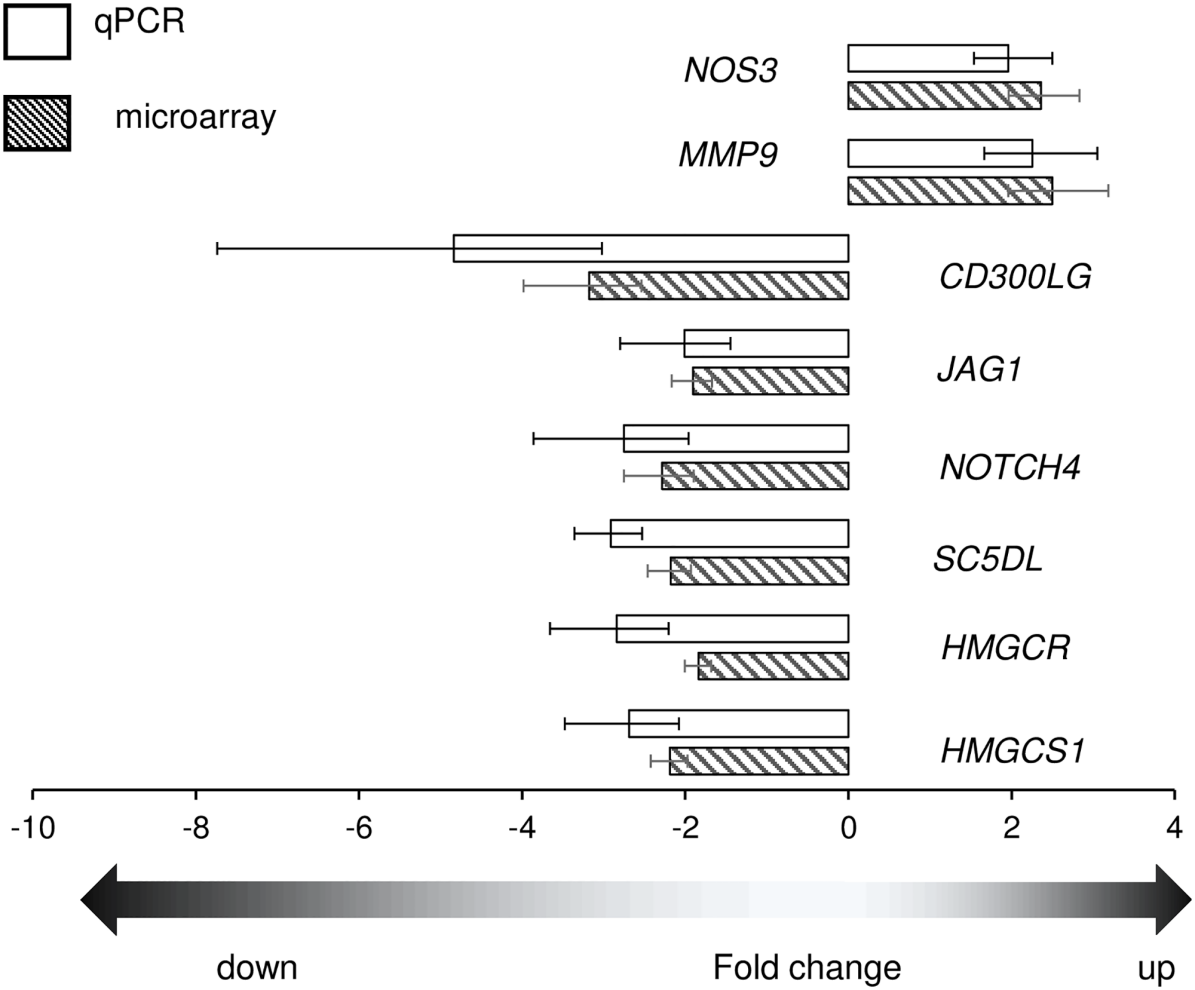
Validation of microarray data with qPCR analysis. Fold change (day 4 vs day 11 CL) of DEG (microarray analyses, dark bars) compared with qPCR results (open bars). Positive and negative values represent up and down-regulated genes, respectively.

**Fig 6:**
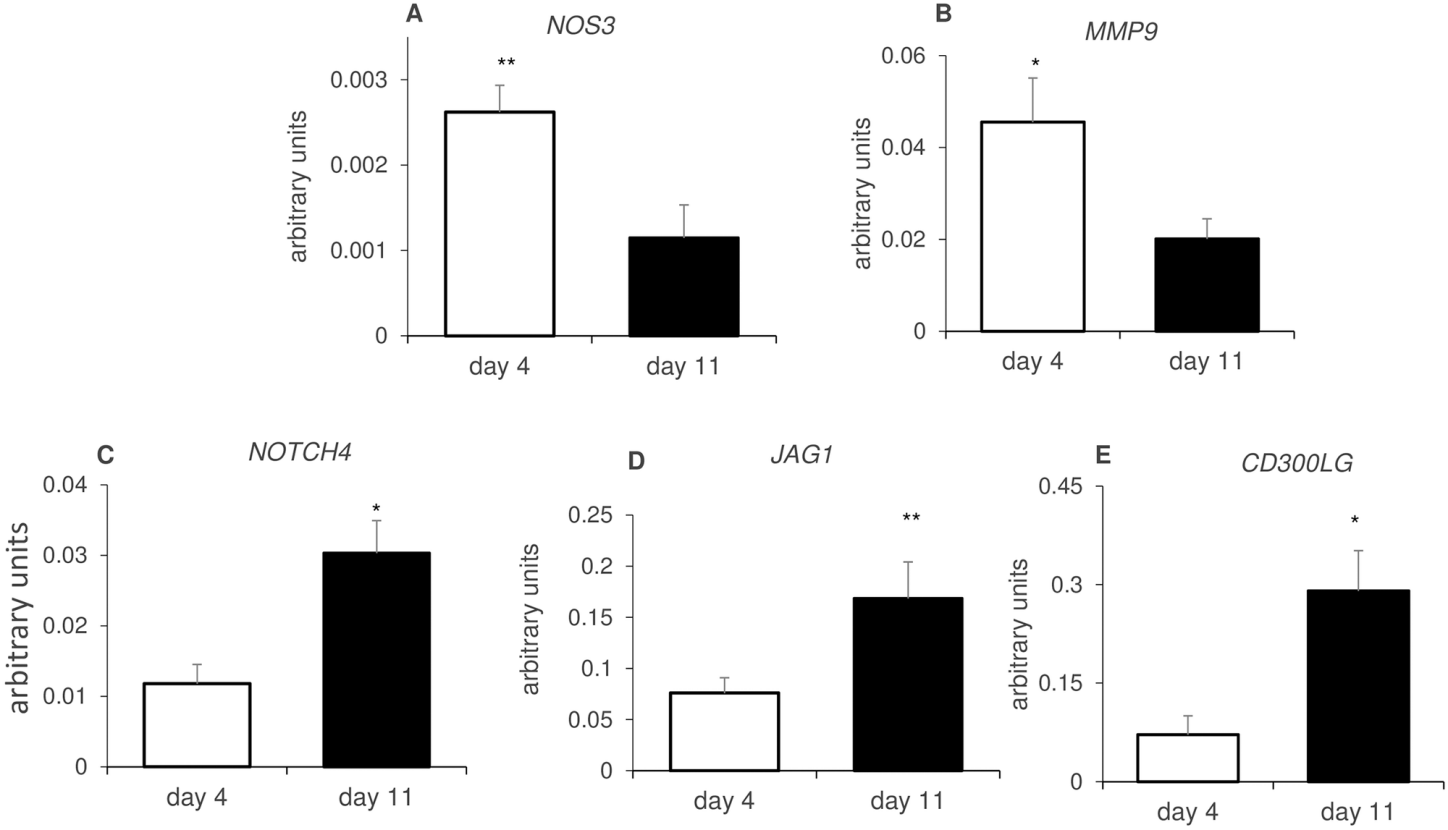
Differential expression of endothelial/blood vessel genes during CL development. mRNA levels of (A) *NOS3*, (B) *MMP9*, (C) *NOTCH4*, (D) *JAG1* and (E) *CD300LG* in the early (day 4) and mid (day 11) luteal stages. Levels of mRNA were measured by qRT-PCR and normalized to *RPS26* in the same samples. The results are presented as means ± SEM. Data were obtained from 5 cows for each luteal stage. Asterisks indicate significant differences between day 4 and day 11; *P<0.05, **P < 0.01, ***P < 0.001. The arbitrary units (Y axis) were multiplied by 100.

**Fig 7:**
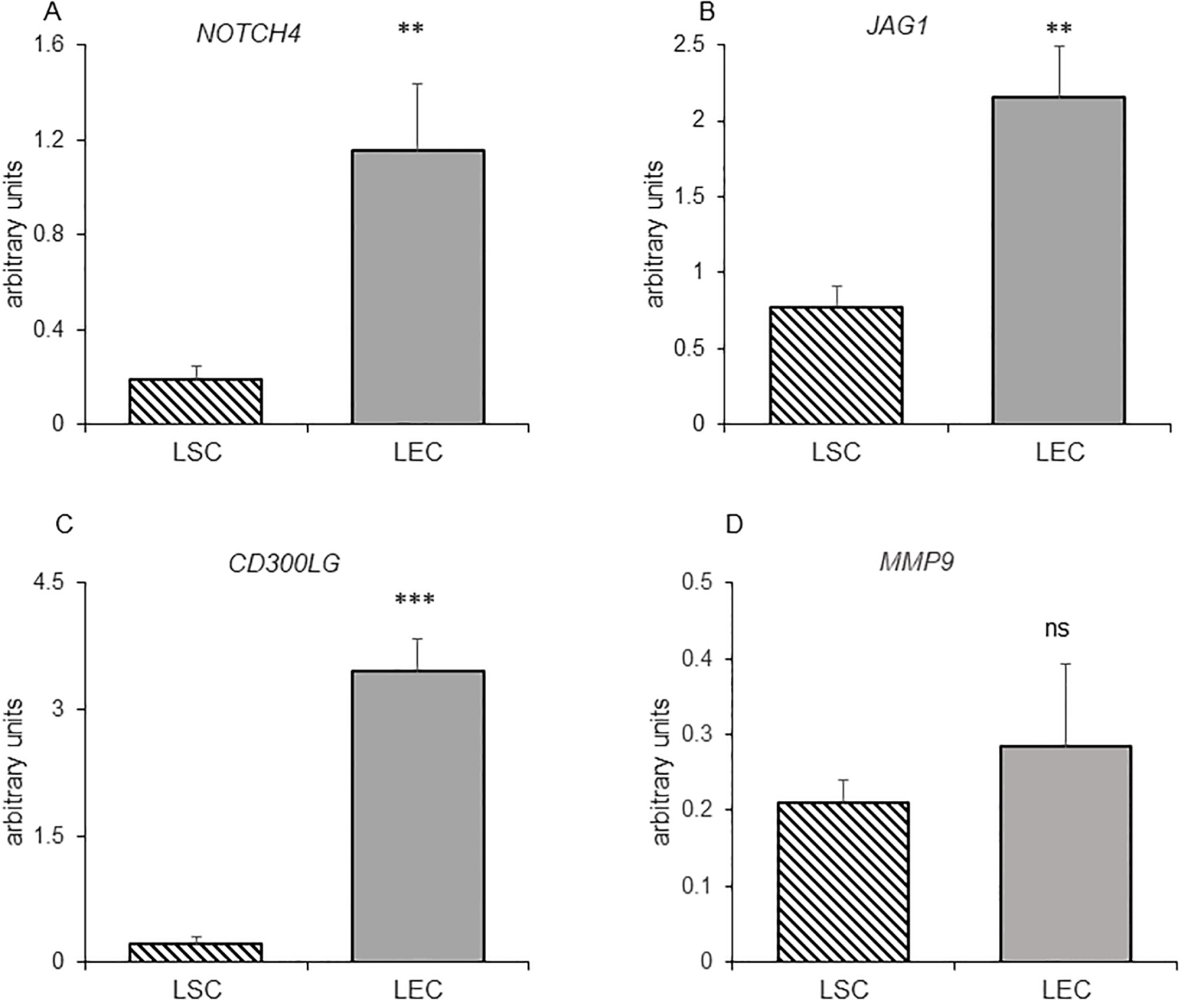
Luteal cell specific expression of *NOTCH4*, *JAG1*, *CD300LG* and *MMP9*. (A) *NOTCH4*, (B) *JAG1* (C) *CD300LG* and (D) *MMP9* mRNA in LSC -luteal steroidogenic cells and LEC–luteal endothelial cells enriched from mid cycle CL. Levels of mRNA were measured by qRT-PCR and normalized to *RPS26* in the same samples. The results are presented as means ± SEM; n = 9. Asterisks indicate significant differences between days 4 and 11; *P<0.05, **P < 0.01, ***P < 0.001. The arbitrary units (Y axis) were multiplied by 100.

Microarray results revealed genes related to endothelial/blood vessel development and function that were highly expressed during the early luteal phase such as: *NOS3* (2.4 fold), *SELE* (4.7 fold), *MMP9* (2.4 fold) while others were highly expressed at mid-luteal stage: *CD300LG* (3.2 fold), *TIE1* (2.1 fold), *NOTCH4* (2.3 fold) and *JAG1* (2 fold). The expression profile of several of these genes were validated by qRT-PCR, it was found that fold changes measured by qPCR were in excellent agreement with microarray results (Fig 5). The mRNA levels of *NOS3* (Fig 6A) and *MMP9* (Fig 6B) were significantly higher (2.3 and 2.3 fold, respectively) on day 4 as compared to day 11, again in accordance with microarray analyses (Fig 5). Oppositely, the mRNA levels of *NOTCH4*, *JAG1* and *CD300LG* (Fig 6C, 6D and 6E) were significantly higher (2.7, 2.4 and 4.1 fold, respectively) in day 11 CL. Then the expression of these genes was determined in two isolated luteal cell types: endothelial and steroiodgenic cells representing major cell populations of the CL. These three genes showed preferential endothelial cell expression, with LEC/LSC ratio of 6.4, 2.8 and 16.4, respectively (Fig 7A–7C). *MMP9* did not show any preferential cell distribution (Fig 7D), while endothelial gene markers *SELE* and *NOS3* were localized to EC, as reported before [33–35].

### Steroidogenic and cholesterol biosynthetic genes upregulated in day 11 vs day 4 CL

**Fig 8:**
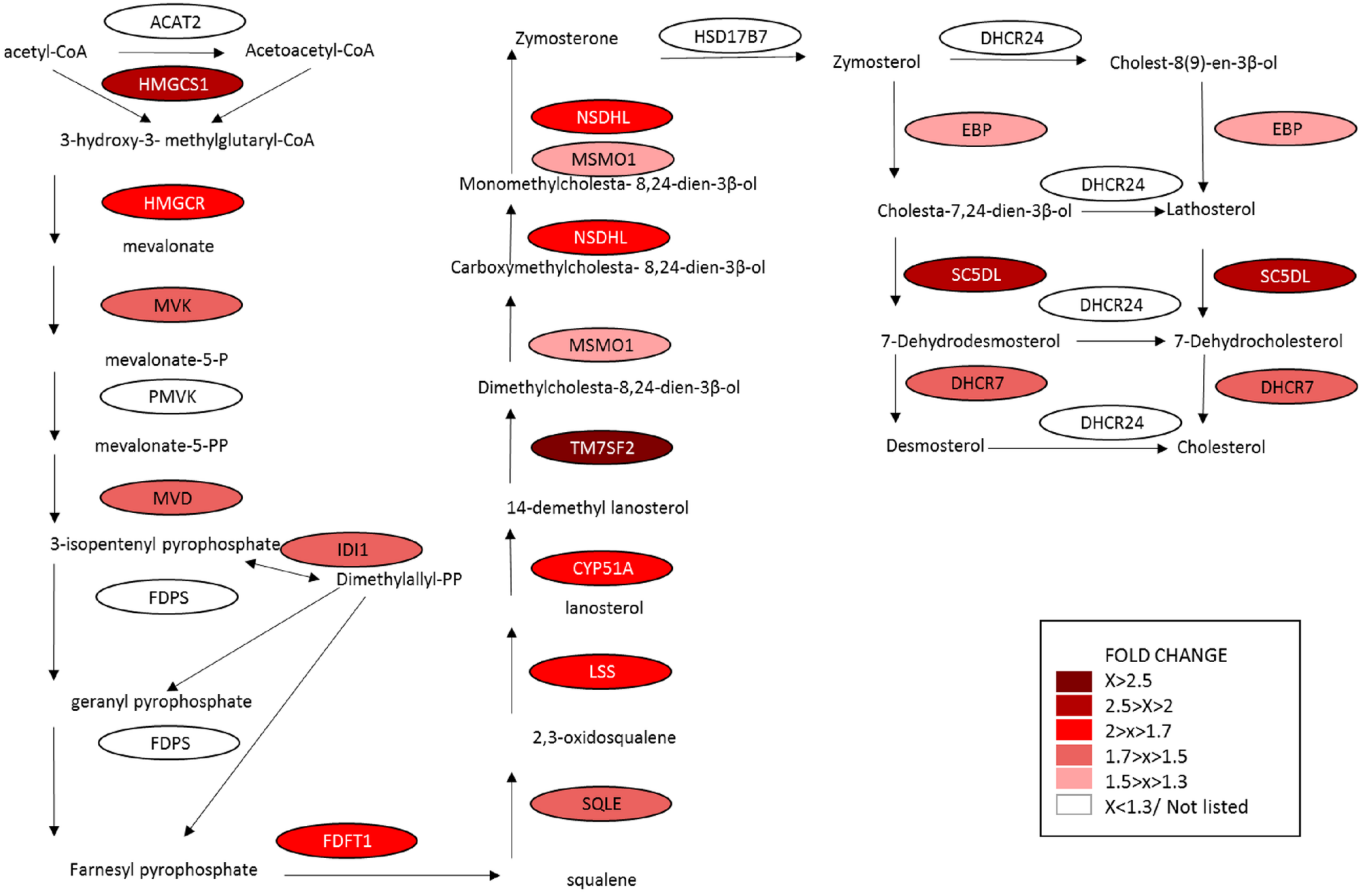
Illustration of differentially expressed cholesterol biosynthetic genes in midcycle CL. The diagram shows up-regulated, DEG (p<0.05) on day 11 vs. day 4 CL. Intensity of the shading increases with the magnitude of the change (see inset).

**Fig 9:**
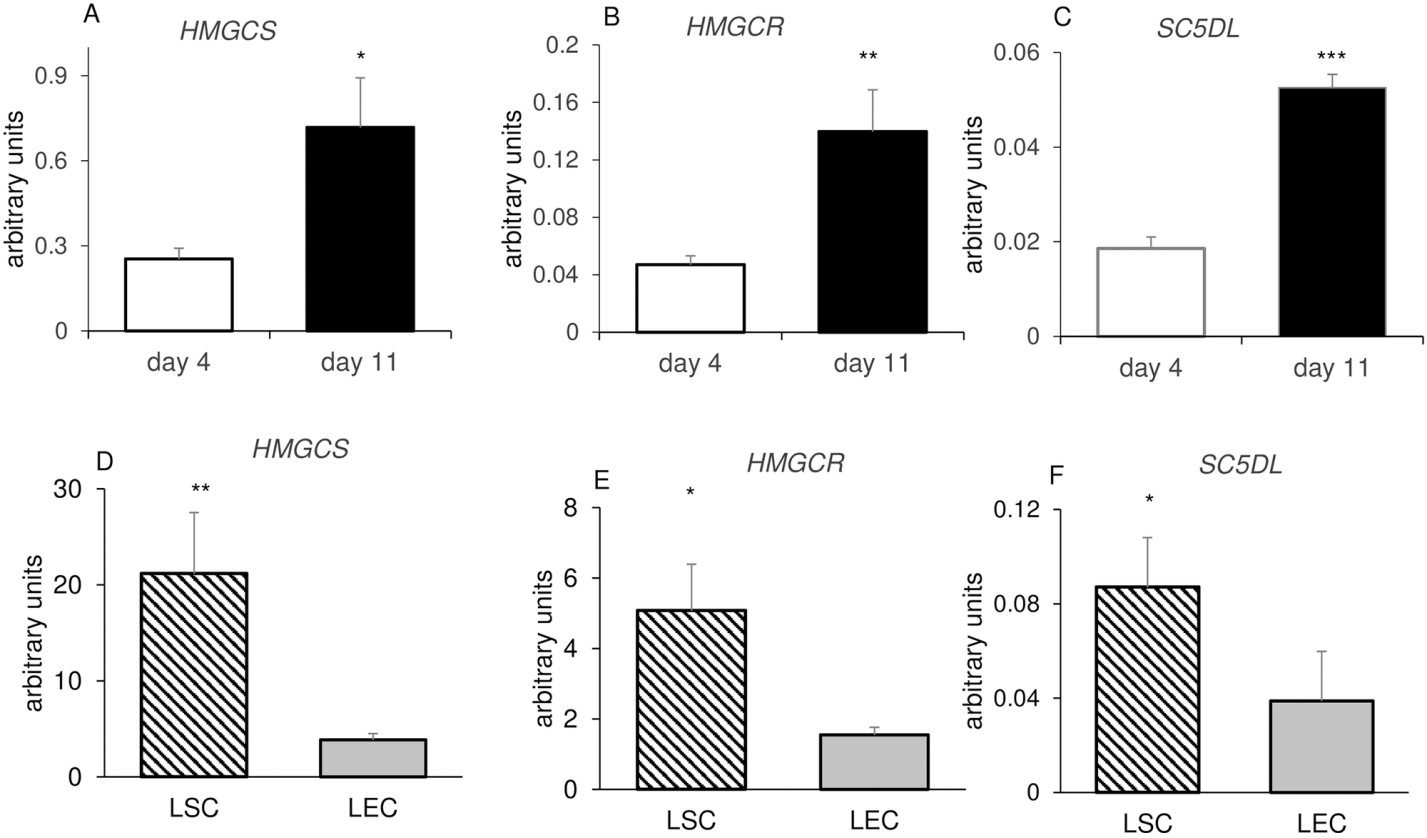
Expression of cholesterol biosynthetic genes in day 4 and 11 CL and their luteal cell specific expression. mRNA expression of (A) *HMGCS*, (B) *HMGCR* and (C) *SC5DL* in the early (day 4) and mid (day 11) luteal stages and cell specific expression of (D) *HMGCS1*, (E) *HMGCR* and (F) *SC5DL* were measured in LEC–luteal endothelial cells and LSC–luteal steroidogenic cells enriched from mid cycle CL. Levels of mRNA were measured by qRT-PCR and normalized to *RPS26* in the same samples. The results are presented as means ± SEM. Data were obtained from 5 cows /CL stage. Asterisks indicate significant differences between day 4 and day 11; *P<0.05, **P < 0.01, ***P < 0.001. The arbitrary units (Y axis) were multiplied by 100.

Data presented in Fig 2 showed significant enrichment of the lipid metabolic process GO term on day 11. We noted that the DEG list on day 11 contained many genes involved in the cholesterol biosynthetic pathway. Some of those genes had 1.5–2 fold difference, therefore, our analysis was expanded to include genes whose fold change was ≥1.5. The cholesterol biosynthetic pathway contains 20 genes [36, 37], 13 of which were upregulated significantly on day 11 vs. day 4 of the cycle based on microarray data analysis (Fig 8). These enzymes include *TM7SF2* (4 fold), *CYP51A1* (1.8 fold), *NSDHL* (1.7 fold), *FDFT1* (1.7 fold), *LSS* (1.7 fold), *SQLE* (1.7 fold), *MVK* (1.7 fold), *MVD* (1.6 fold), *IDI1* (1.6 fold), as well as the rate-limiting enzyme of cholesterol biosynthesis HMG-CoA reductase (*HMGCR* 1.9 fold), the enzyme that catalyzes the first step of cholesterol biosynthesis HMG-CoA synthase (*HMGCS1* 2.2 fold) and Sterol-C5-Desaturase (*SC5DL* 2.2 fold) associated with lethal cholesterol disorders [38]. We verified the expression levels of these last three genes with qPCR as shown in 9A-C. Again, in accordance with microarray analyses *HMGCS*, *HMGCR* and *SC5DL* were significantly higher (2.8, 3.0 and 2.8 fold, respectively) in mid luteal stage (day 11) as compared with the early stage (day 4; Fig 9A–9C). Examining luteal cell-specific expression of *HMGCS*, *HMGCR* and *SC5DL*, we found that these genes were expressed predominantly in luteal steroidogenic cells (Fig 9D–9F) with LSC/LEC ratio of 5.5, 3.3 and 2.2 folds, respectively.

**Fig 10:**
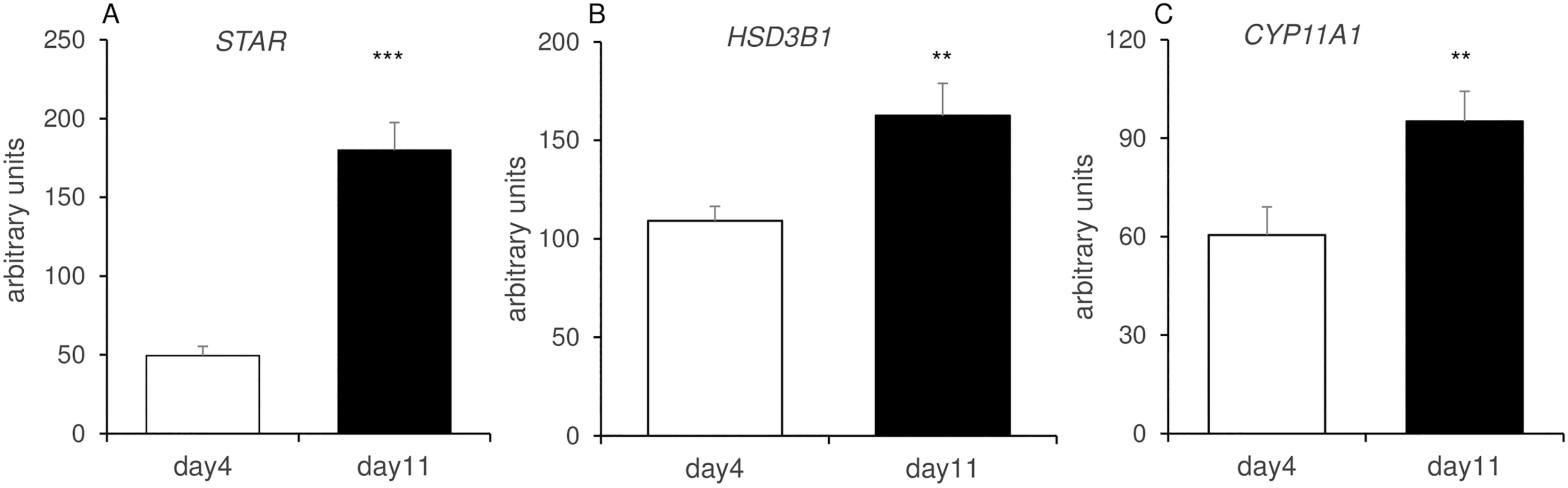
*STAR*, *HSD3B1* and *CYP11A1* expression in day 4 and day 11 CL. mRNA expression of *STAR*, *HSD3B1* and *CYP11A1* (A-C). Levels of mRNA were measured by qRT-PCR and normalized to *RPS26* in the same samples. The results are presented as means ± SEM; n = 9. Asterisks indicate significant difference between day 4 and day 11; *P<0.05, **P < 0.01, ***P < 0.001. The arbitrary units (Y axis) were multiplied by 100.

Next, to examine if the genes involved in cholesterol synthesis are elevated in granulosa cells as a result of luteinazation, cells were cultured in the absence or presence of forskolin (adenylyl cyclase activator) as detailed in Table 2. A significant stimulation of *HMGCS1*, *HMGCR* and *SC5DL* was observed after 24h of forskolin treatment (Table 2). The three main steroidogenic genes (*CYP11A1*, *STAR* and *HSD3B1*) were also determined by qPCR, showing the expected elevation in the mature CL with *STAR* being the most prominently induced, 3.6 fold higher at midcycle as compared with early CL (Fig 10A–10C).

**Table 2:** Effect of 24h treatment with forskolin (10μM) on cholesterol biosynthetic gene expression in granulosa cells.

Gene	Control †	Forskolin †	Significance
***HMGCS1***	3.91±1.05	19.87 ±3.81	*
***HMGCR***	1.78±0.38	6.28±1.59	**
***SC5DL***	0.01±0.002	0.03±0.004	*

The results are presented as means ± SEM.

Data were obtained from 4 different experiments.

Asterisks indicate significant differences from their respective controls;

*P<0.05.

**P < 0.01.

***P < 0.001.

^**†**^ The arbitrary units of gene expression were multiplied by 100.

### Molecular interactions in day 4 and 11 CL

**Fig 11:**
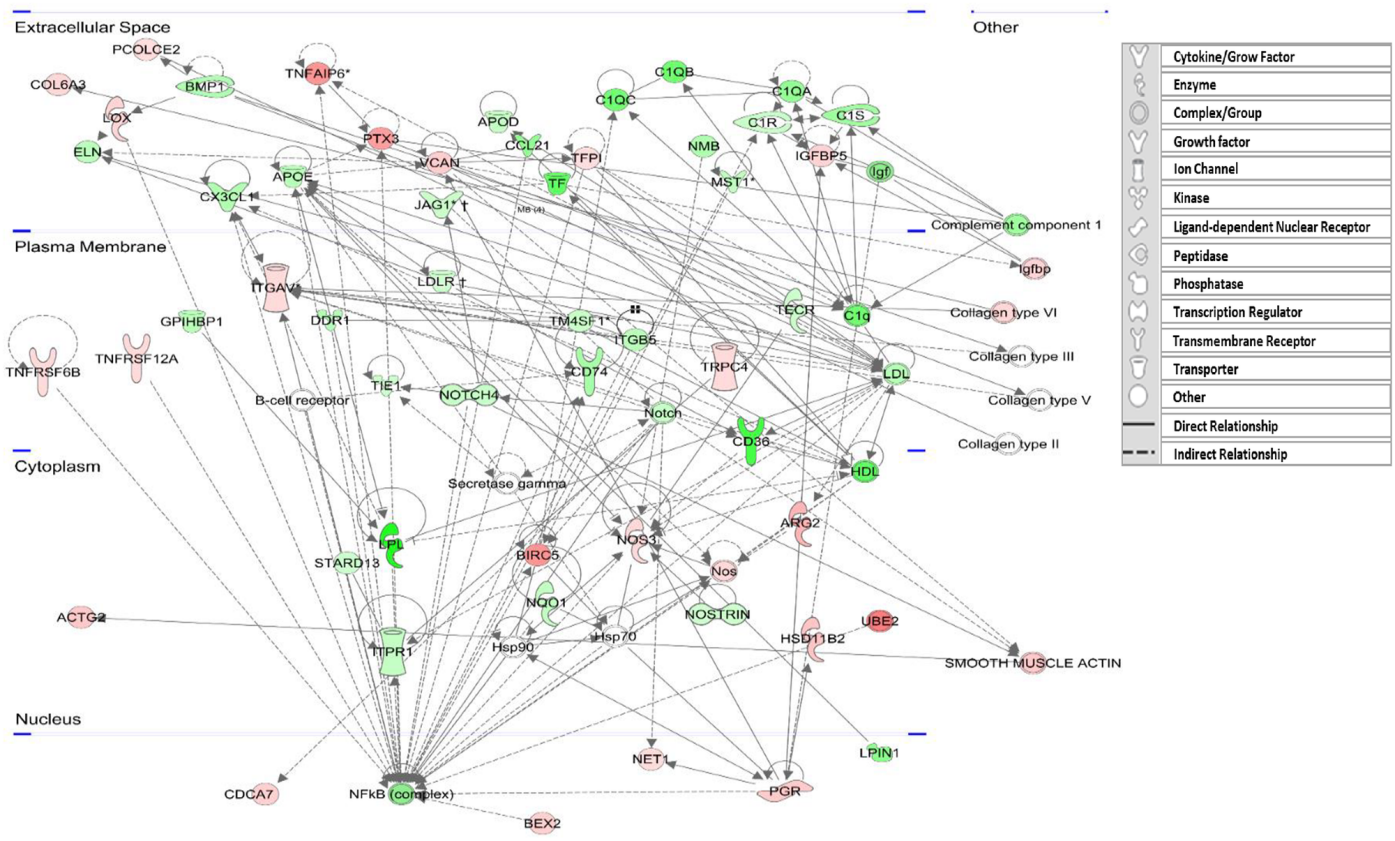
Compound network representing the main underlying biological processes related to day 4 and day 11 CL. Four IPA biological networks [network 6 (score 35), network 8 (score 31), network 10 (score 28) and network 12 (score 26) see S2 File] of the DEG (|fold change ≥2|, P<0.05) between day 4 and day 11 CL were merged and adopted to a larger biological network, indicating the mutual relationship between networks. The network is displayed graphically as nodes (gene/gene products) and edges (the biological relationship between nodes). Red nodes indicate genes that were upregulated in day 4 CL; green nodes indicate genes that were upregulated in day 11 CL. White nodes indicate genes that are not differentially expressed but related to this network. Intensity of the shading increases with the magnitude of the change. † Genes added manually with fold change >1.9.

To further decipher the molecular interactions of the 681 differentially expressed genes, network analysis was performed using the Ingenuity Pathway Analysis (IPA) tool. Twenty five networks were found, 17 of which had 20 or more focus genes in each network (all networks are available in S2 File). Four networks were merged and adapted to form a compound network representing the main underlying biological processes related to CL from these two developmental stages (Fig 11). The compound network shows genes in pivotal positions and the intricate crosstalk between genes and gene complexes. Specifically, the analysis shows NOS complex genes, *IGFBP*, *PTX3-TNFA1P6*, *PGR*, *ACTG2* and *ITGAV* genes being upregulated on day 4 (red). In the mature (day 11) CL, *NOTCH*, *IGF*, *LDL*, *HDL* and NF-κB complexes together with *APOD*, *APOE*, *CD74*, *STARD13*, *CX3CL* and complement system genes were upregulated (green). The compound network strengthens results presented in previous figures.

## Discussion

Using various bioinformatics tools we analyzed the transcriptomes of bovine CL on days 4 and 11. 681 genes (363 and 318 for day 4 and 11, respectively) were differentially expressed. The analysis revealed profound differences in networks, pathways and specific genes expressed in CL on days 4 and 11, representing the early, developing gland vs. the mature bovine CL at its plateau phase.

Previous studies using proliferation markers mainly to the S phase of the cell cycle, such as Bromodeoxyuridine (BrdU), PCNA and Ki67, showed an increased labeling index in the early luteal phase. Using the power of genomic technologies and collection of CL at precise days of cycle we were able to confirm and extend these findings, demonstrating that numerous genes, acting during all phases of the cell cycle (G1, S, G2, and M) were elevated in day 4 versus d 11 CL. These include *CCNB1*, *CCNA1*, *CCNA2*, *CDK1*, *AURKA*, *PLK1* and the MCM complex genes. In fact, DEG on day 4 were greatly dominated by processes such as cell cycle, DNA replication and DNA metabolic process. Most studies comparing early vs. mature CL suggest that the bulk of proliferating cells were LEC or/and pericytes [20, 39, 40].

Our results show that *NOS3* which is involved in maintaining dilated vessels and *MMP9* implicated in the angiogenic process by promoting degradation of the extracellular matrix were elevated on day 4 as compared to day 11. It is noteworthy that *MMP9*, unlike *NOS3*, did not show preferential luteal cell localization, implying that endothelial and non-endothelial cells participate in ECM remodeling. Another novel aspect of the results suggest that endothelial cells on day 11 CL employ a mechanism to prevent further blood vessel sprouting and drive their maturation by expressing *NOTCH4* and *JAG1*. NOTCH4 is a member of the NOTCH transmembrane receptor family that is expressed primarily on endothelial cells. NOTCH signaling is crucial for developmental processes, and is important for many aspects of vascular biology [41]. Using an in vitro endothelial-sprouting assay it was suggested that constitutive NOTCH4 activation in endothelial cells inhibits angiogenesis by various mechanisms [42]. More recently NOTCH4 was discovered to be an inhibitor of the NOTCH1 receptor which is positively correlated with angiogenesis [43]. JAG1 is one of the five notch ligands that include also DLL1, DLL3, DLL4, and JAG2. JAG1 is expressed in endothelial cells (here and [44]) and in vascular smooth muscle cells [44, 45]. It was shown to be indispensable for the development of vascular smooth muscle cells, a fact that may be highly relevant for blood vessel maturation in the CL. Together results presented here suggest that the presence of *NOTCH4* and *JAG1* in blood vessels may inhibit angiogenesis at mid cycle. Previous information suggest a role for Dll4/Notch-1 in luteal blood vessel development [46–50] yet *NOTCH4* and *JAG1* were not studied in CL before. VEGFR-1 (*FLT1*) was also elevated significantly on day 11 (2.3 fold). The negative role of VEGFR-1 in angiogenesis was suggested in several reports [51, 52] reinforcing the idea that VEGFR-1 may act as a VEGF-trap to inhibit the pro-angiogenic VEGFR-2 function. Its expression in day 11 CL may act in concert with JAG1 and NOTCH4 to curb angiogenesis.

Another novel finding from described studies relates to expression of *CD300LG*. While the family of CD300 signaling molecules are detected mostly in myeloid and lymphoid cells, the *CD300LG* gene is exclusively expressed in endothelial cells [53, 54] as also observed here for luteal endothelial cells. The *CD300LG* gene (also known as Nepmucin and CLM-9) encodes for an adhesion molecule that supports L-selectin-dependent lymphocyte migration via its Ig domain. Elevated expression of the *CD300LG* gene at mid cycle would therefore support the transmigration of immune cells into the CL at this stage. Interestingly, monocyte chemoattractant protein 1 **(***MCP-1*/*CCL2*), a chemokine that regulates migration and infiltration of monocytes/macrophages, previously shown to be elevated in day 12 bovine CL [55], was not upregulated in our dataset on day 11. The findings of this study suggest a potential important role for CD300LG in promoting immune cell recruitment into the CL as means to support luteal function and/or in preparation for luteal regression.

In agreement with numerous studies published during last 2 decades, our study detected an abundance of immune related genes, enriched in categories such as regulation of immune system process, complement activation, B cell mediated immunity and acute inflammatory response, in day 11 CL. In addition, immune cell specific marker genes were identified on day 11, both of lymphocytes (*CD74*, *CD52* and *CD151*), and of macrophages (*CD68*, *CD302*).

In cows, plasma progesterone concentrations increase from the early luteal phase and remain high up to mid-late luteal stage [56–58]. CYP11A1, the first rate-limiting enzyme that catalyzes the conversion of cholesterol to pregnenolone, and HSD3B, the enzyme that catalyzes the synthesis of progesterone from pregnenolone [59] were elevated in the mature gland in agreement with previous findings [60, 61]. STAR is crucial for transport of cholesterol to mitochondria and mediates the acute steroidogenic response [62]. We found that it was highly induced at mid cycle CL as also observed as in other studies [61, 63].

However, the findings presented here imply that cholesterol biosynthesis and utilization may constitute complementary mechanism to obtain high progesterone levels. We observed that genes involved in cholesterol biosynthesis pathway and utilization were significantly increased in the day 11 CL. In fact two thirds of the genes in this pathway were elevated in the mid luteal stage, including crucial enzymes for cholesterol production like *HMGCR* (here and also as reported by Rodgers et al, 1987 [17]), *HMGCS* and *SC5DL*. In addition genes encoding proteins for cholesterol uptake and transport from plasma, such as *LDLR*, *APOD* and *APOE* [64, 65] were also significantly enriched in day 11 versus d 4 CL (S1 File). We observed that key cholesterol synthesizing genes (*HMGCR*, *HMGCS* and *SC5DL*) were indeed localized to steroidogenic cells and were responsive to cAMP elevation, further implying their relevance to luteinized steroidogenic cells. Our data therefore strongly suggests that cholesterol synthesis (de novo and from exogenous sources) plays a major role in sustaining luteal progesterone levels. This proposition is supported by the study of Mares et al. [14, 18] in which they showed that luteal cholesterol content was elevated throughout most of the bovine CL lifespan (until day 15).

In summary, this study examined global changes associated with cow’s CL development; it reveals profound differences in networks, pathways and specific genes expressed in the CL. By analyzing the transcriptomes at exact days during CL development and specific luteal cell types, the potential roles of select novel genes were revealed. Only regulation of genes was studied here, the determination of protein products of these genes awaits future research. It is well known that angiogenesis in early CL is enhanced by growth factors and other factors [66]. This study suggests that angiogenesis can also be negatively regulated at midcycle by expressing *JAG1*, *NOTCH4* and *VEGFR-1*. Endothelial gene (*CD300LG*) detected here for the first time in the CL, may play a significant role in immune cell recruitment into the mature CL. Finally, this study suggests the importance of cholesterol availability to the steroidogenically active CL as a means to maintain progesterone synthesis for an extended time during the plateau phase of the cycle.

## Supporting information

S1 File Enriched GO terms in the early (day 4) and mid-luteal (day 11) stages. (XLSX)

S2 File Networks of differentially expressed genes (day 4 vs. 11). (XLSX)
